# The clinical significance of long non-coding RNA ANRIL level in diabetic retinopathy

**DOI:** 10.1007/s00592-019-01442-2

**Published:** 2019-11-06

**Authors:** ShuZe Chen, HuiMin Zhong, Yan Wang, ZiHong Wang, XiaoQian Liang, SiQi Li, ZhenHao Li, ZhengTong Yu, LiYing Li, GuoGuo Yi, Min Fu

**Affiliations:** 1 Department of Ophthalmology, Zhujiang Hospital, Southern Medical University, Guangzhou, Guangdong China; 2 Department of Ophthalmology, Shenzhen Hospital, Southern Medical University, Guangzhou, Guangdong China

**Keywords:** Diabetic retinopathy, Antisense non-coding RNA at the INK4 locus, Renin–angiotensin system, NF-κB pathway, Vascular endothelial growth factor

## Abstract

**Aim:**

To analyse the expression of lncRNA-ANRIL and other related factors in different human body fluids, explore the clinical significance of ANRIL and validate whether ANRIL is interrelated with the renin–angiotensin system and NF-κB signalling pathway.

**Methods:**

Ninety-one patients were included in this cross-sectional study and were divided into the NDM group (20 patients), DM group (25 patients), NPDR group (21 patients) and PDR group (25 patients). Basic information and samples of serum, aqueous fluid and vitreous fluid were collected before vitrectomy or intravitreal injection. The transcription and levels of ANRIL and other related factors were detected by RT-PCR and ELISA. Statistical Package for Social Sciences software was used for statistical analysis.

**Results:**

ANRIL expression varied among different groups and body fluids. There was no difference in ANRIL expression between the NDM and DM groups, but the level of ANRIL was significantly lower in the DM group than in the NPDR and PDR group. In vitreous fluid, ANRIL expression was positively correlated with Ang II, p65 and VEGF expression in the PDR group. The expression of ANRIL in serum was not significantly correlated with age or the random blood sugar but was positively correlated with diabetic duration and HbAc1 level.

**Conclusions:**

Levels of lncRNA-ANRIL are higher in DR patient and correlated with the progression of DR that may be used as an indicator to predict the development of DR. The activation of the RAS and the NF-κB pathway may be closely related to the upregulation of ANRIL.

*Clinical trial number* ChiCTR1800017500. *Registry* Chinese Clinical Trial Registry.

**Electronic supplementary material:**

The online version of this article (10.1007/s00592-019-01442-2) contains supplementary material, which is available to authorized users.

## Introduction

In 2016, more than 400 million people suffered from diabetic mellitus (DM) worldwide, and it is estimated that by 2030, the number of diabetic patients will be as high as 1 billion worldwide. Today, there are approximately 10.2 million diabetic adults aged 40 and over in the USA [1]. Diabetic retinopathy (DR), one of the diseases that seriously endanger human vision and even cause irreversible blindness in the twenty-first century, has become a serious public health problem that cannot be ignored. The approximate prevalence rates of retinopathy and vision-threatening retinopathy are 40.3% and 8.2%, respectively [1].

The pathogenesis of DR is very perplexing because the mechanism of DR is not completely clear. Polyol pathway flux, advanced glycation end product (AGE) formation, protein kinase C (PKC) activation and hexosamine pathway flux constitute the four classical pathways of DR [2]. The oxidative stress theory [3], unified mechanism theory [4] and chronic inflammation theory [5] are frontier research areas in recent years.

In the pathogenesis of DR, a variety of factors are involved in the process of DR lesion development. Long non-coding RNA (lncRNA) is a new discovery in molecular biology research in recent years and is closely related to cell differentiation and individual development [6]. Although preliminary studies have confirmed that a variety of lncRNAs play important roles in the pathogenesis of DR, their mechanisms of action in DR and the mechanism that mediates the pathogenesis of DR have not been fully defined [7].

In 2007, Eric et al. [8] first discovered an antisense non-coding RNA at the INK4 locus in human whole blood leucocytes and lymphoblastoid cell lines in a genetic study of the melanoma–neural tumour syndrome family, named ANRIL (antisense non-coding RNA in the INK4 locus, ANRIL). Genome-wide association studies (GWAS) indicate that ANRIL is a risk locus for diseases such as melanoma, basal cell carcinoma, nasopharyngeal carcinoma, leukaemia, glioma and breast cancer and that it is associated with prostate cancer, gastric cancer and pancreatic cancer [9–11]. In addition, ANRIL is important in the pathogenesis of cardiovascular disease, glaucoma and intracranial aneurysms [12–14].

ANRIL has also been confirmed to be associated with the onset of DR. Overexpression of ANRIL may be a result of the activation of the renin–angiotensin system (RAS) and NF-κB pathway [15–17]. Studies by Thomas et al. [18] showed that ANRIL is a key regulator of VEGF by changing the levels of p300, miR200b and EZH2-mediated PRC2 complexes. After miR200b upregulation, the expression of p300 and PRC2 was significantly inhibited. ANRIL affects the levels of the PRC2 complex and p300 by decreasing the expression of miR200b, and the latter two changes further mediate the pathogenesis of DR [18].

Although the role of ANRIL in DR has been reported in animal and cell experiments, to date, there has been less study on the clinical significance of ANRIL in DR. Therefore, the purpose of this study is to explore the clinical significance of ANRIL in the human body and to verify whether there is an interaction between ANRIL and its related factors, as found in animal and cell experiments, to provide a new basis for the pathogenesis and prediction of DR and provide a new theoretical basis for the treatment of DR.

## Materials and methods

This cross-sectional study was conducted from January 2019 to November 2020 at the Zhongshan Ophthalmic Center at Zhujiang Hospital of Southern Medical University and Southern Hospital of Southern Medical University. The study was performed in accordance with the ethical standards established by the 1964 Declaration of Helsinki and its later amendments, the International Conference on Harmonisation, Good Clinical Practice guidelines and all applicable laws and regulations. The study protocol and one amendment to the protocol were reviewed and approved by the Zhujiang Hospital Human Experimental Committee, and all patients signed informed consent forms.

### Study population and grouping

A total of 91 participants (including 71 patients with DM and 20 patients without DM) were selected from Zhujiang Hospital of Southern Medical University (Guangzhou, China) from January 2019 to November 2020 (Table 1). All participants, whose ages ranged from 20 to 80 years, were in need of either vitrectomy or intravitreal drug injection. Patients with other types of retinopathy, such as eye infection, trauma, tumour or other vascular disease of the fundus; patients with medication interference; pregnant or breastfeeding women, and so on, were excluded; 71 Diabetics patients were diagnosed according to the 2018 diabetes diagnosis and treatment standards from the American Diabetes Association (ASA) and were divided into three groups: the DM, NPDR and PDR groups. The DM group comprised 25 patients with DM but without DR. The NPDR group comprised 21 patients with non-proliferative diabetic retinopathy (NPDR). The PDR group comprised 25 patients with proliferative diabetic retinopathy (PDR). Additionally, the control group (NDM group) consisted of 20 participants without DM. Random blood glucose measurement, glycated haemoglobin content measurement, parallel ophthalmoscopy and FFA assessment of the patient’s condition were conducted for all participants, who offered their personal information, including sex, age, primary disease status, duration of diabetes and high blood pressure status. This study was approved by the Ethics Committee of Zhujiang Hospital of Southern Medical University. All patients had signed informed consent forms.

**Table 1:** Subject inclusion and grouping

Group	Disease	Number of patients	Treatment after sample collection
NDM	Cataract	11	IOL implantation + vitrectomy
	IMEM	5	Anterior membrane dissection
	RRD	2	Vitrectomy
	High myopia	2	IOL implantation
DM	Cataract	18	IOL implantation + vitrectomy
	IMEM	4	Anterior membrane dissection
	RRD	3	Vitrectomy
NPDR	Cataract	14	IOL implantation + vitrectomy
	Macular oedema	7	Triamcinolone acetonide injection
PDR	Cataract	6	IOL implantation + vitrectomy
	PDR in V phase	4	Vitrectomy
	PDR in VI phase	15	Vitrectomy

*IMEM* idiopathic macular epiretinal membrane, *RRD* rhegmatogenous retinal detachment, *IOL* intraocular lens

### Sample collection

#### Collection of peripheral blood samples

Peripheral serum samples were obtained preoperatively. Peripheral blood was collected and sent to the laboratory immediately for the measurement of various indicators.

#### Collection of aqueous humour

Aqueous humour was collected before vitreous injection or vitrectomy. After routine disinfection, towel spreading and eyelid opening, the conjunctival sac was disinfected with iodophor solution diluted to 0.5 g/l and washed repeatedly with sterile saline. Then, the effusion in the conjunctival sac was removed with sterile gauze. A sterile 1-ml disposable plastic TB syringe was connected with a 9-gauge needle, and anterior chamber puncture was performed within 1 mm of the corneal limbus. Within 2 to 5 s, the anterior chamber fluid was extracted from the central pupil area without touching the iris, lens and corneal endothelial cells, and 0.2 ml of undiluted anterior chamber fluid was obtained. The specimens were moved to a sterilized and silicified 5-ml Eppendorf tube, placed into liquid nitrogen and stored in a refrigerator at − 70 to − 80 °C until analysis.

#### Collection of vitreous fluid

After routine disinfection, towel spreading and three applications of topical anaesthesia, the eyelids were opened with an eyelid opener. At a distance of 3.5 mm behind the limbus of the cornea, a 4.5 needle was inserted into the vitreous body at the 12:00 position. A 0.3 ml volume of vitreous body fluid was withdrawn from the liquefied area of the vitreous body and centrifuged immediately at 12,000 r/min for 10 min. The supernatant was taken as the vitreous body specimen and stored in a refrigerator at − 80°C.

### ELISA

An ELISA was performed to measure the protein expression of AngII, AT1R, p65, p52 and VEGF using a commercially available kit (ALPCO, Salem, NH, USA; R&D Systems, Minneapolis, MN, USA) according to the manufacturer’s instructions.

### RT-PCR

Total RNA was extracted from serum, aqueous humour and vitreous humour using Trizol reagent (Invitrogen; Thermo Fisher Scientific, Inc.). RNA quality was assessed using a NanoDrop™ 2000 spectrophotometer (Thermo Fisher Scientific, USA), and samples with an A260/A280 ratio between 1.8 and 2.0 were used for reverse transcription to synthesize cDNA. The cDNA synthesis conditions were as follows: 42 °C for 60 min, 95 °C for 10 min, termination of the reaction and storage at − 40 °C. The expression levels of ANRIL, AngII, AT1R, p65, p52, VEGF and the reference gene GAPDH were measured by qRT-PCR. cDNA was added to a real-time PCR system. The reagents were mixed and centrifuged and were then subjected to real-time PCR. By analysis of the CT values, the results were compared with the expression of the reference gene GAPDH by the relative threshold cycle method. The primer sequences are shown in Table 2.

**Table 2:** Amplification primer sequences

Primer	Upstream	Downstream
ANRIL	5′-TTATGCTTTGCAGCACACTGG-3′	5′-GTTCTGCCACAGCTTTGATCT-3′
mRNA-AngII	5′-CTGCAAGGATCTTATGACCTGC-3′	5′-TACACAGCAAACAGGAATGGGC-3′
mRNA-AT1R	5′-GGCCAGTGTTTTTCTTTTGAATTTAGCAC-3′	5′-TGAACAATAGCCAGGTATCGATCAATGC-3′
mRNA-VEGF	5′-GCTCTCTTGGGTGCACTGGA-3′	5′-CACCGCCTTGGCTTGTCACA-3′
mRNA-p65	5′-ATCCCATCTTTGACAATCGTGC-3′	5′-CTGGTCCCGTGAAATACACCTC-3′
mRNA-p52	5′-AGAGGCTTCCGATTTCGATATGG-3′	5′-GGATAGGTCTTTCGGCCCTTC-3′
GAPDH	5′-ACAGCAACAGGGTGGTGGAC-3′	5′-TTTGAGGGTGCAGCGAACTT-3′

### Statistical analysis

Data were analysed with Statistical Package for Social Sciences version 24.0 software (SPSS Inc., Chicago, IL, USA). The descriptive statistics are presented as the means ± SDs for normally distributed continuous variables. Nonnumerical data are presented as percentages. Each of the samples was measured twice, and the average was used as the result. Once the coefficient of variation in the sample is more than 5%, we consider that there is an error in the result of one measurement. The third measurement was added, and calculate the CV value again. If the CV value is still greater than 5%, this sample will be given up. If the CV value is smaller than 5%, take the average of these three as the result, or take the average of the other two as the result after eliminating the data which obviously deviate from the mean. Normally distributed data were compared by using the F-test and the t-test, and nonparametric tests were used for data that did not conform to a normal distribution. Comparisons among multiple groups were performed by LSD and SNK (*q* test) tests. A linear regression and the Spearman rank correlation test were used to evaluate the interaction between different indicators. Finally, *P* < 0.05 was considered statistically significant in all analyses.

## Results

### Characteristics of the participants in this study

A total of 91 participants (aged 35–85) were included in the analysis (Table 3). Age significantly differed between groups being highest in the PDR group (*P* = 0.002). Also, markedly difference was observed in random blood glucose level (*P* < 0.001) and HbA1c level (*P* < 0.001). However, no difference was seen between sex and eyesight (*P* > 0.05) or between DM duration and hypertension (*P* > 0.05).

**Table 3:** Characteristics of the participants in this study

Index	NDM	DM	NPDR	PDR	*P* value
Total people, *n* (%)	20 (22.0%)	25 (27.5%)	21 (23.1%)	25 (27.5%)	–
Sex, *n* (%)					
Female	10 (50.0%)	13 (52.0%)	8 (38.1%)	13 (52.0%)	0.760
Male	10 (50.0%)	12 (48.0%)	13 (61.9%)	12 (48.0%)	
Age (years)	55.70 ± 10.06	59.48 ± 9.03	59.19 ± 9.373	65.00 ± 9.768	0.015
Eyesight					
Left	0.12 ± 0.20	0.19 ± 0.23	0.30 ± 0.53	0.39 ± 0.73	0.670
Right	0.26 ± 0.45	0.14 ± 0.20	0.10 ± 0.13	0.38 ± 0.56	0.253
DM duration (years)	–	9.12 ± 7.93	10.33 ± 6.36	11.52±6.08	0.060
Random blood glucose (mmol/l)	5.86 ± 1.82	10.55 ± 1.98	9.46 ± 2.63	11.28 ± 2.35	< 0.001
HbA1c (% mmol/mol)	5.40 ± 0.87	7.72 ± 1.23	10.58 ± 2.42	10.58 ± 2.47	< 0.001
Hypertension, *n* (%)					
Yes	7 (35.0%)	13 (52.0%)	10 (47.6%)	15 (60.0%)	0.411
No	13 (65.0%)	12 (48.0%)	11 (52.4%)	10 (40.0%)	

Categorical variables, including sex and hypertension status, were compared by using the Chi-squared test; continuous variables, such as age and random blood glucose level, were compared by using one-way analysis of variance (ANOVA). The Jonckheere–Terpstra test was used for eyesight, DM duration and HbA1c level

### The expression of lncRNA-ANRIL and other relevant factors

**Fig. 1:**
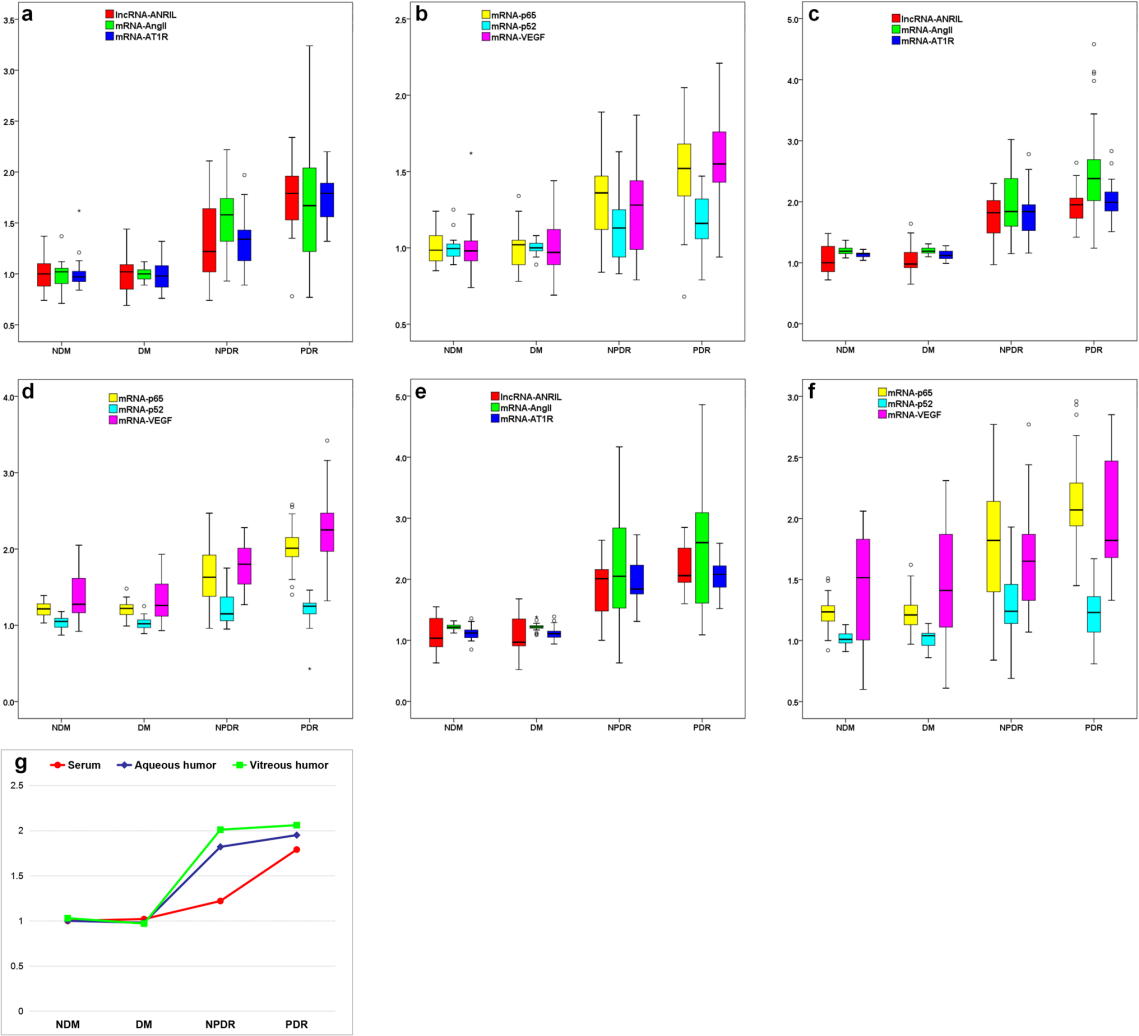
Expression of lncRNA-ANRIL and other relevant factors in serum, aqueous humour and vitreum. **a** Expression of lncRNA-ANRIL, mRNA-AngII and mRNA-AT1R in serum among different groups. **b** Expression of mRNA-p65, mRNA-p52 and mRNA-VEGF in serum among different groups. **c** Expression of lncRNA-ANRIL, mRNA-AngII and mRNA-AT1R in aqueous humour among different groups. **d** Expression of mRNA-p65, mRNA-p52 and mRNA-VEGF in aqueous humour among different groups. **e** Expression of lncRNA-ANRIL, mRNA-AngII and mRNA-AT1R in vitreous humour among different groups. **f** Expression of mRNA-p65, mRNA-p52 and mRNA-VEGF in vitreous humour among different groups. **g** Median expression of lncRNA-ANRIL in different fluids. With the expression of GAPDH gene as a reference, the CT values of various indicators from different sources of body fluids and different groupings were expressed by box diagram. Different detection factors are expressed by different colours. The ordinate is the relative expression content of each factor. Box diagrams show the median, upper quartile (75%), lower quartile (25%), upper and lower limit of different gene expression. “°” indicates the discrete value of the data, and “*” indicates the extreme value of the data

Relative expression of ANRIL, mRNA-Ang II, mRNA-AT1R, mRNA-p65, mRNA-p52 and mRNA-VEGF varied in serum, aqueous humour and vitreous humour (Fig. 1). The expression of ANRIL in all three body fluids was highest in PDR, while NDM and DM groups had similar values and NPDR group showing intermediate values. Expression of ANRIL, mRNA-AngII, mRNA-AT1R, mRNA-p65 and mRNA-VEGF showed similar trend in all three body fluids (*P* ≤ 0.005), while mRNA-p52 expression in all three body fluids did not differ between the groups (*P* > 0.05).

Concentrations of the proteins related to ANRIL in all three body fluids are shown in Table 4. The trend in these proteins was similar to that in the mRNA. However, serum p52 showed a significant difference across groups (being highest in the PDR group, *P* < 0.05).

**Table 4:** Concentration of AngII, AT-IR, VEGF, p65 and p52 in serum aqueous humour and vitreous humour in the four groups

Factor	Sample source	NDM	DM	NPDR	PDR
Ang II (pg/ml)	Serum*	18.3 ± 5.6	28.1±6.8	33.0 ± 9.3	43.2 ± 11.0
	Aqueous humour*	102.3 ± 32.2	103.7 ± 19.5	234.1 ± 69.3	257.4 ± 63.5
	Vitreous humour*	112.6 ± 30.6	115.0 ± 16.2	232.2 ± 72.1	303.5 ± 116.7
AT-1R (pg/ml)	Serum*	2.4 ± 0.5	3.3±0.6	3.6 ± 0.7	4.7 ± 1.3
	Aqueous humour*	2.2 ± 0.4	2.2 ± 0.5	6.2 ± 1.2	6.6 ± 1.3
	Vitreous humour*	2.4 ± 0.7	2.2 ± 0.6	6.5 ± 0.9	7.6 ± 1.3
VEGF (pg/ml)	Serum*	459.2 ± 100.7	498.8 ± 92.6	575.4±103.1	664.5 ± 128.5
	Aqueous humour*	411.0 ± 57.0	422.5 ± 56.9	725.1 ± 99.6	806.5 ± 204.8
	Vitreous humour*	462.0 ± 81.5	482.9 ± 81.8	717.8 ± 93.2	874.3 ± 148.3
p65 (μg/l)	Serum*	1.0 ± 0.2	1.0 ± 0.1	1.1 ± 0.2	1.6 ± 0.3
	Aqueous humour*	1.1 ± 0.2	1.1 ± 0.2	1.1 ± 0.2	1.3 ± 0.3
	Vitreous humour*	1.1 ± 0.2	1.2 ± 0.2	1.3 ± 0.2	1.5 ± 0.4
p52 (μg/l)	Serum*	0.6 ± 0.1	0.5 ± 0.1	0.6 ± 0.1	0.8 ± 0.2
	Aqueous humour	0.6 ± 0.1	0.6 ± 0.1	0.5 ± 0.1	0.8 ± 0.1
	Vitreous humour	0.7 ± 0.1	0.6 ± 0.1	0.5 ± 0.10	0.8 ± 0.1

“*” denotes groups with significant differences (*P* < 0.05) after Jonckheere–Terpstra test

### Comparison of the expression of lncRNA-ANRIL in different body fluids and different groups

The expression levels of ANRIL in different body fluids were significantly different. Further analysis showed that there was no obvious difference between the expression of ANRIL level in different body fluids in the NDM and DM groups (all *P* > 0.05). In the NPDR group, the level of ANRIL in serum was significantly different from that in aqueous humour and vitreous humour (both *P* < 0.05), but the ANRIL level in the vitreous humour was slightly higher (but not significantly) than that in the aqueous humour (*P* = 0.471). However, in the PDR group, there was no significant difference in the ANRIL level between serum and aqueous humour (*P* = 0.186), while there was a clear difference between aqueous humour and vitreous fluid (*P* = 0.012) or serum and vitreous fluid (*P* < 0.001).

Further analysis showed that the expression levels of ANRIL in different groups were different. The expression of ANRIL among NDM and DM groups was similar regardless of any body fluid source (both *P* > 0.05), but the ANRIL level in the NPDR group was significantly higher than that in the DM group (all *P* < 0.05, regardless of the body fluid source). The ANRIL level in the peripheral blood was much higher in the PDR group than in the NPDR group (*P* = 0.002), but there was no such significant difference for the aqueous humour (*P* = 0.251) and vitreous humour (*P* = 0.052) (Fig. 1g).

### Relationship between ANRIL, RAS, NF-κB and VEGF in vitreous humour

**Fig. 2:**
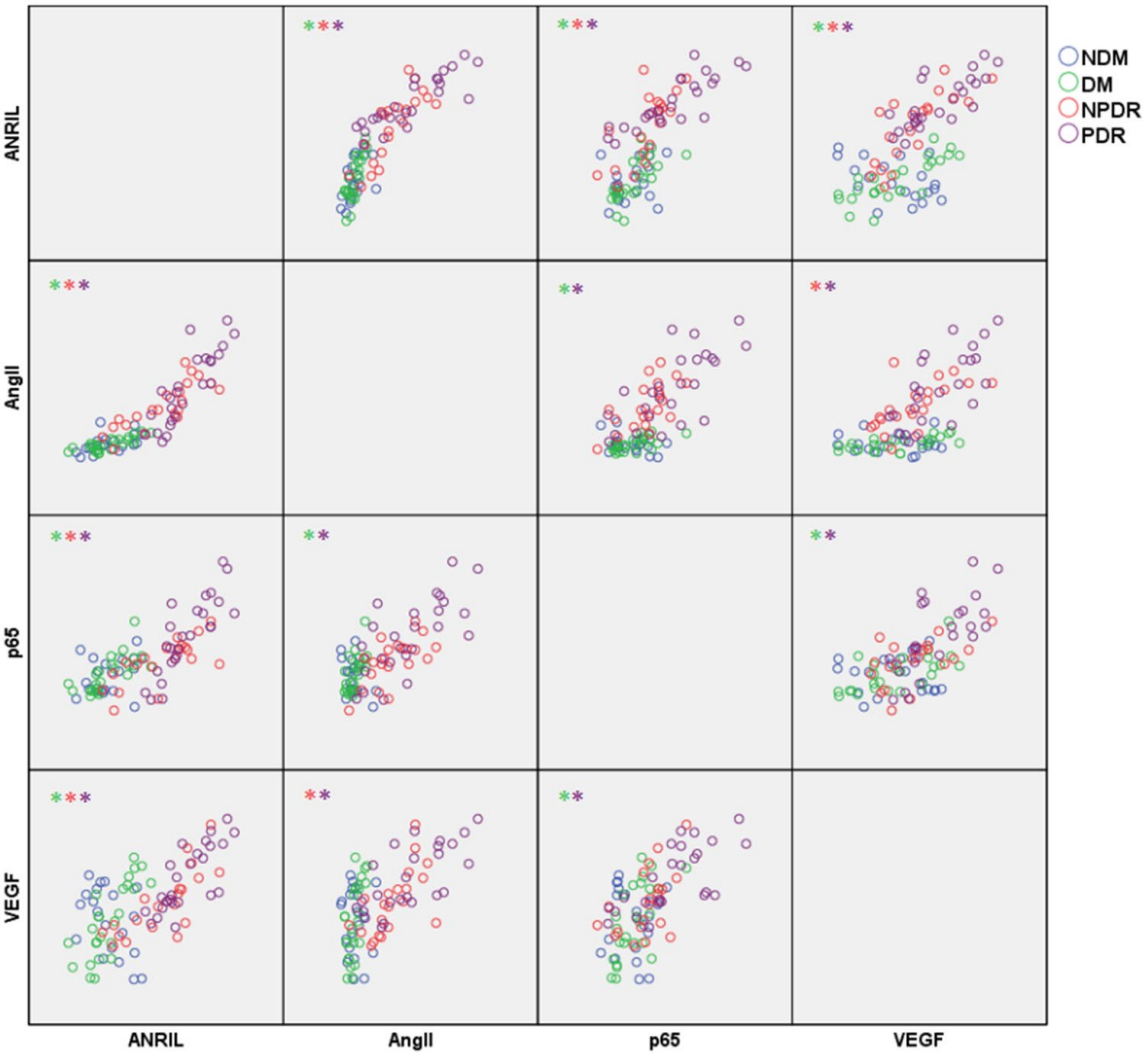
Correlation analysis matrix scatter plot between the expression of ANRIL, AngII, p65 and VEGF. The “*” in the upper left corner of each box denotes a group with a linear correlation and a *ρ* value greater than 0.7. The different colours represent the different groups

We further analysed the correlation among lncRNA-ANRIL, the mRNA of AngII, p65 and VEGF in vitreous fluid from each group; the scatter plot is shown in Fig. 2.

In the NDM group, no correlation was observed between the various indicators. After Spearman correlation analysis, in the DM group, a substantially linear trend was observed between ANRIL and AngII (*P* < 0.001, *ρ* = 0.792), ANRIL and VEGF (*P* < 0.001, *ρ* = 0.798), and AngII and p65 (*P* = 0.001, *ρ* = 0.654). There was a clear positive correlation between these factors. In the NPDR group, there was a significant positive correlation between ANRIL and AngII (*P* = 0.01, *ρ* = 0.893), ANRIL and p65 (*P* = 0.002, *ρ* = 0.695), AngII and p65 (*P* = 0.004, *ρ* = 0.594), AngII and VEGF (*P* < 0.001, *ρ* =  0.721), and ANRIL and VEGF (*P* = 0.004, *ρ* =  0.594). In the PDR group, a linear trend existed between ANRIL and AngII (*P* = 0.001, *ρ* = 0.863), ANRIL and p65 (*P* = 0.005, *ρ* = 0.837), ANRIL and VEGF (*P* = 0.001, *ρ* = 0.802), AngII and p65 (*P* < 0.001, *ρ* = 0.699), and p65 and VEGF (*P* = 0.01, *ρ* = 0.754).

### Relationship between ANRIL in peripheral blood and other indicators in different groups

**Fig. 3:**
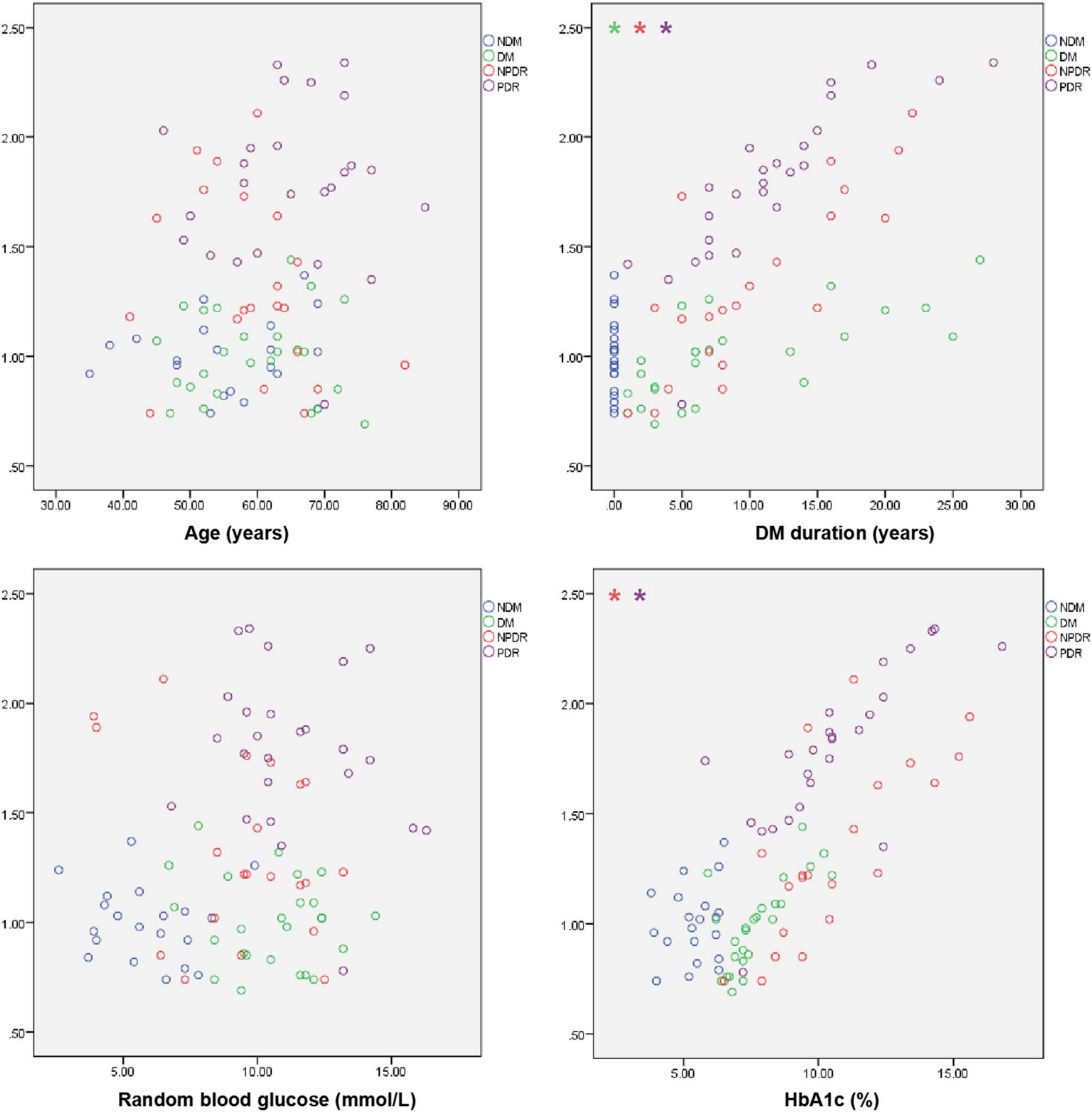
Correlation analysis between ANRIL in peripheral blood and age, diabetes duration, random blood glucose level and glycosylated haemoglobin level. The “*” in the upper left corner of each box denotes a group with a linear correlation and a *ρ* value greater than 0.7. The different colours represent the different groups and the ordinates represent the relative expression of lncRNA-ANRIL

Further analysis of the blood ANRIL level and age, diabetes duration, random blood glucose level, HbAc1 level, and hypertension status in the different subgroups showed that there was no significant correlation between ANRIL and these baseline indicators in the NDM group. In the DM group, there was a significant correlation between the duration of diabetes (*P* = 0.010, *ρ* = 0.742) and the ANRIL level, and the longer the duration of diabetes, the higher was the relative level of ANRIL in peripheral blood. In the NPDR group, the relative level of ANRIL in peripheral blood increased significantly with the prolongation of diabetes (*P* = 0.050, *ρ* = 0.796) and the increase in glycated haemoglobin (*P* = 0.001, *ρ* = 0.770). This trend was more pronounced in the NPDR group for patients with PDR (DM duration: *P* = 0.001, *ρ* = 0.932; HbAc1: *P* = 0.005, *ρ* = 0.83) (Fig. 3).

## Discussion

Diabetic retinopathy is a severe diabetic complication and is currently the leading cause of blindness worldwide, but its pathogenesis is not yet clear, and its therapeutic effect is not promising [19]. According to Siddiqui A et al., lncRNA-ANRIL might be a genetic predictor for DR [20]. Anu Alice Thomas et al. revealed that ANRIL expression can be upregulated in vascular endothelial cells through PRC2, miR200b and p300 and that ANRIL can mediate the proliferation of retinal vascular endothelial cells and participate in the pathogenesis of DR [18]. However, whether ANRIL follows the same process in the human body has not yet been reported. To indirectly verify the relationship between ANRIL and DR, this study analysed the difference in the levels of ANRIL and its related factors among non-diabetic, diabetic, NPDR and PDR patients from three body fluid sources: human peripheral blood, aqueous humour and the vitreous body.

Our study found that there were significant differences in the expression of ANRIL among different groups regardless of the body fluid source (*P* < 0.001). Further comparisons revealed that there was no significant difference in the ANRIL level between the NDM and DM groups (*P* > 0.05), suggesting that ANRIL was not characteristically expressed in DM patients. The expression of ANRIL in the NPDR group was significantly higher than that in the DM group, while the difference between the ANRIL level in the vitreous and aqueous humours was more significant. However, the expression of vitreous and serum-derived ANRIL in the NPDR group was significantly different from that in the PDR group, but ANRIL expression in aqueous humour did not differ, suggesting that the composition of aqueous humour does not completely represent the composition of vitreous humour. Further correlation analysis showed that the expression of ANRIL in different body fluids increased with the progression of DR. In conclusion, ANRIL may be a predictor of DR, and its expression is positively correlated with the severity of DR. However, because this study was a cross-sectional study, it did not follow the changes in ANRIL expression in a specific individual over an extended course of DR and thus ignored human heterogeneity. Therefore, further prospective studies need to be implemented to explore deeper mechanisms.

We found that the ANRIL level in serum was significantly lower than that in aqueous humour and the vitreous body in all groups, while in the NPDR group, the vitreous-derived ANRIL level was higher than the aqueous humour-derived ANRIL level in the PDR group, suggesting that the distribution of ANRIL in different body fluids is unequal, and the closer the source to the ocular lesions, the higher the ANRIL expression was. We are curious about what causes this difference between aqueous humour and the vitreous body. On the one hand, we cannot rule out the possibility of sample contamination in the experiment. On the other hand, although aqueous humour and the vitreous body are interlinked, aqueous humour is produced from scleral venous sinuses and is not completely mixed with colloid vitreous fluid; hence, the content of aqueous humour is not the same as that of the vitreous body, and ANRIL is no exception [21].

ANRIL has been shown to be associated with diabetes and diabetic complications. Zhou X et al. found that the expression of ANRIL was related to coronary heart disease in diabetic patients. The expression of ANRIL in the peripheral blood of patients with coronary heart disease was significantly higher than that in patients without coronary heart disease. This result suggested that ANRIL could be used as a potential peripheral biomarker [16]. In Zhang B’s study, overexpressed lncRNA-ANRIL upregulated vascular endothelial growth factor and increased angiogenesis by activating the NF-κB signalling pathway in diabetic rats with cerebral infarction [15]. As mentioned above, ANRIL, as a key regulator of vascular endothelial growth factor, plays an important role in the pathogenesis of DR. Vascular endothelial growth factor (VEGF) promotes retinal neovascularization by stimulating the growth of RVECs, leading to the occurrence of PDR. VEGF is currently the target of many drugs for DR [22]. ANRIL is also expected to become a breakthrough point in DR treatment.

Although the role of ANRIL in regulating vascular endothelial growth factor (VEGF) in the pathogenesis of DR has been explained in detail, to date, no studies have reported the reasons for the upregulation of ANRIL in DR patients. According to Kanda A et al., hyperglycaemia can activate the renin–angiotensin system in the retinal microenvironment. AngII can act on the AT1R receptor on retinal vascular endothelial cells and activate the intracellular NF-κB pathway [17]. Zhou et al. [16] showed that activated p65 molecules in RVECs could bind to the Chr9p21 region and induce the transcription of lncRNA-ANRIL. It can be inferred that the activation of the RAS and NF-κB in the retina is involved in the upregulation of ANRIL. Our study showed a linear positive correlation between AngII and ANRIL in vitreous fluid (*P* < 0.001, *ρ* = 0.792) and a similar correlation between p65 and ANRIL (*P* = 0.005, *ρ* = 0.837). In addition, there was a correlation between AngII and p65 (*P* = 0.001, *ρ* = 0.654). These associations were particularly evident in the PDR group but almost nonexistent in the normal control group. This pattern indirectly verifies the interaction between the RAS, p65 and ANRIL in the retina and their joint participation in the pathogenesis of DR.

We further analysed the relevance between the basic information of the patients and the level of ANRIL in serum. Peripheral blood-derived ANRIL expression was not related to age but was associated with the course of diabetes mellitus and the level of glycosylated haemoglobin, and this association was significantly stronger in the PDR group than in the other groups. On the one hand, this result shows that ANRIL expression does not increase with age. On the other hand, because the activation of ANRIL depends on the RAS and NF-κB, with the development of diabetes, the activation of NF-κB and the RAS increases, which leads to upregulation of ANRIL expression. Glycated haemoglobin effectively reflects the blood sugar control of diabetic patients in the past 1–2 months and, to some extent, reflects the severity of diabetes. Our results suggest that the level of ANRIL in peripheral blood increases with the increase in HbA1c, which may also be due to the upregulation of ANRIL expression mediated by hyperglycaemia.

In conclusion, our study shows that lncRNA-ANRIL is higher in retinopathy groups being highest in PDR group. This suggests that lncRNA-ANRIL may be used as a potential index to predict the development of DR. The levels of expression of AngII, p65 and ANRIL are significantly correlated. Combined with previous experimental studies, our results suggest that activation of the local RAS and the NF-κB pathway may play an important role in the upregulation of ANRIL. Since this study was a cross-sectional study without any intervention or long-term observation of patients, further studies need to be carried out to understand the mechanism of ANRIL in human DR.

## Electronic supplementary material

Below is the link to the electronic supplementary material.

Supplementary material 1 (DOCX 100 kb)
