# Supplementary material for: The clinical significance of long non-coding RNA ANRIL level in diabetic retinopathy
Source: Acta Diabetol. 2019 Nov 6;57(4):409–18. doi: 10.1007/s00592-019-01442-2 (PMC7093365; doi:10.1007/s00592-019-01442-2)
Supplement: Supplementary file 1 — Supplementary material 1 (DOCX 100 kb) [file 592_2019_1442_MOESM1_ESM.docx]

| **The coefficient of variation value of all indicators from different body fluid by ELASA and PCR** | | | | | | | | | | | | | | | | | | | | | | | | | | | | | | | | | | | | | | | |
| --- | --- | --- | --- | --- | --- | --- | --- | --- | --- | --- | --- | --- | --- | --- | --- | --- | --- | --- | --- | --- | --- | --- | --- | --- | --- | --- | --- | --- | --- | --- | --- | --- | --- | --- | --- | --- | --- | --- | --- |
| **Number** | **Group** | **ELASA result** | | | | | | | | | | | | | | | | |  | **PCR result** | | | | | | | | | | | | | | | | | | | |
|  |  | **Serum** | | | | |  | **Aqueous humour** | | | | |  | **Vitreous humour** | | | | |  | **Serum** | | | | | |  | **Aqueous humour** | | | | | |  | **Vitreous humour** | | | | | |
|  |  | **AngII** | **AT1R** | **p65** | **p52** | **VEGF** |  | **AngII** | **AT1R** | **p65** | **p52** | **VEGF** |  | **AngII** | **AT1R** | **p65** | **p52** | **VEGF** |  | **AngII** | **AT1R** | **p65** | **p52** | **VEGF** | **ANRIL** |  | **AngII** | **AT1R** | **p65** | **p52** | **VEGF** | **ANRIL** |  | **AngII** | **AT1R** | **p65** | **p52** | **VEGF** | **ANRIL** |
| 1 | NDM | 0.00% | 4.21% | 3.31% | 1.74% | 2.38% |  | 1.24% | 4.35% | 4.38% | 0.54% | 1.40% |  | 4.99% | 3.46% | 4.24% | 4.69% | 0.32% |  | 2.12% | 3.26% | 3.12% | 3.40% | 3.63% | 3.16% |  | 4.13% | 3.96% | 4.16% | 3.30% | 4.84% | 3.86% |  | 4.52% | 4.18% | 4.19% | 3.76% | 4.79% | 3.70% |
| 2 | NDM | 2.18% | 2.13% | 2.22% | 1.46% | 4.88% |  | 0.91% | 3.46% | 3.67% | 1.32% | 2.78% |  | 3.08% | 3.08% | 4.89% | 0.69% | 1.13% |  | 3.02% | 3.81% | 3.45% | 3.49% | 3.16% | 3.45% |  | 4.50% | 3.91% | 4.45% | 3.49% | 4.48% | 3.00% |  | 4.06% | 3.77% | 4.05% | 3.72% | 4.79% | 4.15% |
| 3 | NDM | 0.13% | 0.00% | 2.87% | 1.07% | 0.01% |  | 4.97% | 3.08% | 3.40% | 0.66% | 0.78% |  | 1.94% | 1.16% | 4.99% | 4.13% | 3.38% |  | 3.67% | 3.67% | 3.85% | 3.67% | 3.40% | 3.89% |  | 4.17% | 4.22% | 4.63% | 3.85% | 3.94% | 3.15% |  | 4.20% | 4.77% | 4.55% | 3.44% | 3.04% | 3.61% |
| 4 | NDM | 4.00% | 3.08% | 4.69% | 1.37% | 4.44% |  | 4.71% | 1.65% | 3.26% | 2.95% | 0.14% |  | 0.30% | 0.02% | 3.26% | 1.44% | 3.29% |  | 4.05% | 3.21% | 3.54% | 3.63% | 2.87% | 4.05% |  | 4.42% | 4.18% | 3.98% | 4.02% | 4.78% | 2.17% |  | 4.59% | 4.67% | 4.84% | 3.54% | 5.00% | 3.20% |
| 5 | NDM | 4.35% | 4.76% | 3.49% | 0.65% | 4.95% |  | 0.06% | 4.65% | 4.56% | 0.26% | 3.38% |  | 4.69% | 0.16% | 3.45% | 0.99% | 2.78% |  | 3.02% | 3.16% | 3.72% | 3.54% | 2.27% | 2.37% |  | 4.60% | 3.86% | 3.67% | 3.81% | 3.47% | 4.81% |  | 4.69% | 3.77% | 4.97% | 3.98% | 2.76% | 4.45% |
| 6 | NDM | 2.82% | 3.36% | 4.47% | 0.69% | 0.72% |  | 4.96% | 4.76% | 4.89% | 0.63% | 1.52% |  | 4.98% | 4.59% | 2.47% | 4.10% | 0.10% |  | 3.67% | 3.59% | 3.02% | 4.17% | 3.76% | 2.67% |  | 4.04% | 4.41% | 4.45% | 3.76% | 4.56% | 4.67% |  | 4.33% | 3.73% | 4.62% | 4.10% | 1.58% | 4.91% |
| 7 | NDM | 1.74% | 0.31% | 2.67% | 0.87% | 4.50% |  | 3.97% | 2.13% | 3.72% | 4.96% | 0.85% |  | 1.21% | 4.98% | 3.36% | 2.95% | 4.98% |  | 3.26% | 4.09% | 4.05% | 3.40% | 3.93% | 3.67% |  | 4.04% | 3.77% | 4.84% | 3.54% | 4.99% | 3.49% |  | 4.37% | 3.47% | 3.52% | 3.11% | 4.55% | 3.15% |
| 8 | NDM | 0.05% | 4.82% | 3.26% | 1.74% | 4.99% |  | 0.11% | 1.89% | 4.69% | 0.14% | 2.03% |  | 4.29% | 1.68% | 4.27% | 0.05% | 4.98% |  | 3.40% | 4.38% | 3.93% | 3.21% | 3.93% | 4.53% |  | 4.80% | 3.96% | 4.12% | 3.35% | 3.22% | 4.96% |  | 4.41% | 3.53% | 3.69% | 3.35% | 3.90% | 2.61% |
| 9 | NDM | 1.11% | 4.78% | 3.31% | 1.55% | 4.30% |  | 0.48% | 4.21% | 3.31% | 4.64% | 4.80% |  | 0.54% | 5.00% | 4.38% | 3.29% | 0.06% |  | 3.59% | 3.40% | 2.82% | 3.16% | 3.45% | 3.63% |  | 4.30% | 4.18% | 4.21% | 3.81% | 4.56% | 3.59% |  | 4.49% | 2.83% | 4.19% | 3.44% | 4.96% | 4.91% |
| 10 | NDM | 0.35% | 1.46% | 3.63% | 1.69% | 0.13% |  | 2.86% | 0.15% | 2.77% | 4.44% | 3.29% |  | 3.38% | 4.82% | 4.69% | 0.46% | 4.99% |  | 3.72% | 2.77% | 4.47% | 3.02% | 4.41% | 3.76% |  | 4.46% | 4.09% | 4.60% | 3.76% | 4.93% | 3.15% |  | 4.66% | 4.67% | 4.55% | 3.76% | 3.38% | 4.93% |
| 11 | NDM | 1.75% | 0.00% | 4.74% | 0.91% | 4.30% |  | 4.00% | 3.17% | 3.49% | 4.64% | 4.87% |  | 0.07% | 0.59% | 3.36% | 4.77% | 4.17% |  | 3.16% | 3.21% | 4.41% | 3.26% | 3.21% | 4.13% |  | 4.30% | 4.18% | 4.63% | 3.30% | 4.83% | 3.86% |  | 4.24% | 4.41% | 4.19% | 3.40% | 3.70% | 4.11% |
| 12 | NDM | 4.01% | 2.13% | 4.05% | 1.11% | 4.50% |  | 3.89% | 2.38% | 3.54% | 0.07% | 0.00% |  | 0.72% | 0.16% | 4.53% | 4.96% | 1.81% |  | 3.02% | 2.97% | 3.45% | 3.63% | 4.99% | 2.27% |  | 4.42% | 4.22% | 4.25% | 3.20% | 4.59% | 2.69% |  | 4.15% | 4.30% | 4.52% | 3.81% | 4.23% | 3.65% |
| 13 | NDM | 4.50% | 0.04% | 3.40% | 1.46% | 0.37% |  | 0.27% | 4.51% | 3.63% | 3.89% | 1.48% |  | 0.00% | 5.00% | 3.40% | 4.92% | 0.03% |  | 2.72% | 3.21% | 3.63% | 3.59% | 2.67% | 3.16% |  | 4.46% | 3.72% | 4.53% | 4.18% | 3.16% | 4.44% |  | 4.37% | 3.87% | 4.88% | 3.89% | 4.28% | 3.41% |
| 14 | NDM | 4.58% | 4.84% | 3.36% | 1.23% | 0.95% |  | 0.41% | 5.00% | 4.13% | 3.70% | 3.58% |  | 0.52% | 0.29% | 4.69% | 1.44% | 1.40% |  | 3.49% | 3.40% | 3.16% | 3.76% | 2.57% | 3.36% |  | 4.57% | 4.26% | 4.49% | 3.85% | 3.93% | 4.07% |  | 4.29% | 4.14% | 4.98% | 3.44% | 1.64% | 5.00% |
| 15 | NDM | 4.23% | 2.03% | 3.45% | 2.17% | 0.00% |  | 0.16% | 4.94% | 3.26% | 4.82% | 0.13% |  | 0.01% | 0.36% | 4.72% | 0.57% | 0.95% |  | 4.80% | 3.63% | 2.97% | 3.31% | 3.21% | 3.67% |  | 3.89% | 4.22% | 4.02% | 4.06% | 4.67% | 2.89% |  | 4.45% | 4.18% | 3.18% | 3.40% | 4.50% | 4.58% |
| 16 | NDM | 3.94% | 3.46% | 3.02% | 1.78% | 0.06% |  | 2.82% | 0.69% | 3.49% | 4.20% | 4.97% |  | 4.77% | 4.84% | 2.92% | 1.73% | 3.46% |  | 3.93% | 3.49% | 3.21% | 3.63% | 3.63% | 4.47% |  | 4.17% | 4.30% | 4.34% | 2.90% | 4.48% | 2.58% |  | 4.49% | 3.73% | 4.36% | 3.58% | 4.22% | 1.75% |
| 17 | NDM | 0.00% | 0.02% | 3.26% | 1.83% | 2.38% |  | 2.00% | 4.82% | 3.67% | 0.44% | 4.36% |  | 2.64% | 4.93% | 3.45% | 0.22% | 3.89% |  | 3.81% | 4.99% | 4.31% | 3.36% | 3.45% | 4.80% |  | 4.30% | 4.18% | 4.60% | 4.06% | 4.48% | 2.17% |  | 4.33% | 3.92% | 4.40% | 4.02% | 4.97% | 2.01% |
| 18 | NDM | 3.66% | 1.20% | 3.36% | 1.41% | 5.00% |  | 1.12% | 1.46% | 3.63% | 4.93% | 4.07% |  | 1.64% | 4.53% | 3.76% | 1.44% | 3.58% |  | 3.76% | 3.45% | 2.82% | 4.50% | 3.12% | 2.77% |  | 4.34% | 4.26% | 4.66% | 4.29% | 4.70% | 4.67% |  | 4.37% | 3.96% | 4.52% | 3.49% | 4.77% | 2.45% |
| 19 | NDM | 1.75% | 2.63% | 3.31% | 2.32% | 4.62% |  | 3.88% | 2.13% | 4.41% | 2.16% | 3.46% |  | 4.38% | 0.20% | 4.27% | 2.16% | 4.98% |  | 3.67% | 3.12% | 3.16% | 3.16% | 3.72% | 2.52% |  | 4.30% | 4.22% | 4.07% | 3.63% | 4.60% | 4.77% |  | 4.56% | 4.14% | 4.59% | 3.58% | 4.51% | 4.96% |
| 20 | NDM | 2.74% | 0.13% | 2.42% | 1.46% | 0.95% |  | 1.66% | 4.24% | 3.49% | 4.10% | 1.40% |  | 4.99% | 1.56% | 4.67% | 3.78% | 1.52% |  | 4.01% | 3.07% | 3.49% | 3.67% | 3.59% | 3.31% |  | 4.26% | 4.18% | 4.02% | 3.54% | 3.83% | 2.84% |  | 4.49% | 4.14% | 4.40% | 3.63% | 4.93% | 2.93% |
| 21 | DM | 0.88% | 0.06% | 2.77% | 1.25% | 3.92% |  | 0.02% | 3.94% | 4.99% | 1.85% | 2.78% |  | 1.94% | 3.55% | 4.44% | 4.26% | 0.72% |  | 3.02% | 3.72% | 3.31% | 3.67% | 3.26% | 2.97% |  | 4.13% | 3.96% | 4.53% | 3.39% | 4.35% | 4.44% |  | 4.33% | 3.68% | 3.90% | 3.35% | 4.06% | 3.41% |
| 22 | DM | 4.99% | 1.63% | 2.98% | 1.02% | 0.03% |  | 2.01% | 3.17% | 3.26% | 4.97% | 0.34% |  | 1.06% | 2.93% | 4.17% | 2.91% | 1.06% |  | 3.31% | 4.09% | 2.97% | 3.72% | 3.67% | 3.40% |  | 4.30% | 3.91% | 4.02% | 3.58% | 4.99% | 4.98% |  | 3.91% | 3.63% | 3.63% | 3.72% | 4.87% | 3.09% |
| 23 | DM | 0.18% | 0.16% | 3.18% | 1.12% | 2.51% |  | 4.33% | 2.58% | 3.45% | 4.33% | 0.08% |  | 0.05% | 2.68% | 4.27% | 0.10% | 0.06% |  | 3.67% | 3.36% | 3.72% | 3.54% | 4.47% | 3.63% |  | 4.50% | 4.18% | 3.98% | 3.85% | 4.52% | 3.20% |  | 4.49% | 4.01% | 4.55% | 3.81% | 4.96% | 4.07% |
| 24 | DM | 4.95% | 0.01% | 3.27% | 1.07% | 4.69% |  | 3.59% | 4.57% | 4.38% | 0.15% | 5.00% |  | 3.08% | 1.65% | 4.86% | 4.96% | 0.13% |  | 3.93% | 3.45% | 4.74% | 3.63% | 2.47% | 2.87% |  | 4.66% | 4.22% | 4.66% | 4.18% | 4.72% | 3.00% |  | 4.82% | 4.18% | 4.36% | 3.40% | 4.55% | 4.91% |
| 25 | DM | 3.47% | 0.02% | 3.74% | 1.17% | 0.05% |  | 0.07% | 4.31% | 4.17% | 0.01% | 3.21% |  | 1.64% | 4.24% | 3.31% | 4.20% | 4.98% |  | 3.63% | 3.67% | 2.52% | 3.89% | 2.03% | 3.85% |  | 4.08% | 3.77% | 4.80% | 4.49% | 4.52% | 3.44% |  | 4.29% | 4.70% | 4.99% | 3.25% | 3.46% | 3.25% |
| 26 | DM | 3.79% | 0.99% | 4.14% | 0.75% | 0.85% |  | 0.03% | 3.12% | 3.31% | 0.03% | 4.82% |  | 0.54% | 1.98% | 3.36% | 0.17% | 0.02% |  | 3.76% | 2.82% | 3.26% | 3.45% | 4.91% | 3.16% |  | 4.42% | 4.41% | 4.16% | 3.35% | 4.48% | 3.35% |  | 4.24% | 4.84% | 4.19% | 4.02% | 2.71% | 3.15% |
| 27 | DM | 4.99% | 2.71% | 3.24% | 0.54% | 4.07% |  | 1.94% | 2.13% | 3.63% | 0.15% | 4.03% |  | 2.60% | 4.99% | 3.72% | 0.75% | 0.17% |  | 3.63% | 2.37% | 3.02% | 3.02% | 3.67% | 4.69% |  | 3.99% | 4.30% | 3.72% | 3.00% | 4.38% | 2.89% |  | 4.49% | 3.77% | 4.55% | 3.76% | 4.66% | 2.88% |
| 28 | DM | 0.43% | 3.01% | 2.54% | 0.81% | 0.10% |  | 1.08% | 4.28% | 3.36% | 4.92% | 2.73% |  | 4.29% | 4.99% | 4.76% | 0.09% | 1.85% |  | 3.45% | 3.26% | 3.67% | 3.26% | 4.05% | 3.93% |  | 4.38% | 3.91% | 3.50% | 3.63% | 4.05% | 3.15% |  | 4.45% | 3.73% | 4.99% | 3.98% | 4.32% | 4.45% |
| 29 | DM | 1.80% | 2.73% | 3.73% | 1.58% | 2.38% |  | 4.64% | 3.36% | 2.87% | 3.92% | 3.50% |  | 1.32% | 3.98% | 4.72% | 0.08% | 3.74% |  | 3.49% | 3.85% | 3.76% | 3.81% | 4.41% | 4.44% |  | 4.46% | 3.86% | 4.96% | 3.49% | 4.35% | 4.11% |  | 4.37% | 3.96% | 4.19% | 3.01% | 4.00% | 4.77% |
| 30 | DM | 0.14% | 1.11% | 4.14% | 1.14% | 3.85% |  | 0.01% | 4.90% | 3.63% | 1.85% | 4.57% |  | 2.69% | 2.58% | 4.50% | 1.81% | 0.88% |  | 3.21% | 3.02% | 4.05% | 3.45% | 3.40% | 2.82% |  | 4.26% | 4.26% | 3.72% | 3.76% | 4.16% | 2.69% |  | 4.56% | 3.92% | 4.62% | 2.86% | 3.04% | 4.61% |
| 31 | DM | 2.82% | 0.06% | 3.39% | 1.66% | 0.30% |  | 1.06% | 3.46% | 3.67% | 0.63% | 0.95% |  | 0.52% | 4.84% | 3.36% | 0.66% | 4.20% |  | 3.16% | 4.41% | 3.21% | 3.49% | 3.02% | 2.27% |  | 4.26% | 4.00% | 4.07% | 4.10% | 4.59% | 3.63% |  | 4.37% | 4.18% | 4.14% | 3.81% | 4.00% | 3.09% |
| 32 | DM | 2.74% | 0.26% | 4.39% | 0.60% | 3.92% |  | 2.25% | 0.40% | 4.38% | 2.38% | 2.29% |  | 4.96% | 0.31% | 3.49% | 3.04% | 0.08% |  | 3.72% | 3.93% | 2.92% | 3.36% | 3.45% | 3.45% |  | 4.22% | 4.05% | 4.29% | 3.30% | 3.54% | 3.90% |  | 4.41% | 4.14% | 3.41% | 3.89% | 4.96% | 4.07% |
| 33 | DM | 0.85% | 4.27% | 3.06% | 0.67% | 3.74% |  | 4.22% | 2.13% | 3.02% | 1.32% | 2.20% |  | 0.99% | 4.09% | 3.67% | 3.42% | 4.93% |  | 3.85% | 2.92% | 2.47% | 3.63% | 3.63% | 4.91% |  | 4.66% | 4.18% | 4.60% | 3.20% | 3.78% | 4.23% |  | 3.96% | 3.77% | 4.62% | 3.72% | 3.96% | 4.81% |
| 34 | DM | 4.29% | 4.64% | 2.52% | 1.05% | 0.57% |  | 1.80% | 3.17% | 3.31% | 4.20% | 0.88% |  | 3.04% | 3.55% | 3.81% | 2.29% | 3.38% |  | 4.05% | 3.49% | 3.81% | 3.59% | 3.36% | 4.38% |  | 4.26% | 4.45% | 4.77% | 3.30% | 4.56% | 4.59% |  | 4.37% | 4.18% | 4.99% | 3.54% | 4.42% | 4.91% |
| 35 | DM | 2.08% | 0.06% | 3.44% | 1.53% | 1.60% |  | 1.44% | 1.03% | 3.72% | 0.10% | 1.44% |  | 0.06% | 4.10% | 3.16% | 0.09% | 4.30% |  | 3.16% | 3.89% | 3.67% | 3.54% | 4.27% | 2.37% |  | 4.13% | 4.58% | 4.66% | 3.85% | 3.94% | 3.40% |  | 4.49% | 3.53% | 4.19% | 4.14% | 4.55% | 4.94% |
| 36 | DM | 4.94% | 0.13% | 3.51% | 1.74% | 0.03% |  | 0.72% | 0.41% | 4.89% | 1.24% | 3.85% |  | 0.54% | 0.38% | 4.89% | 2.69% | 0.01% |  | 3.40% | 4.69% | 2.92% | 3.36% | 4.74% | 2.27% |  | 4.50% | 3.72% | 4.16% | 3.76% | 4.95% | 3.63% |  | 4.45% | 4.18% | 4.10% | 3.30% | 4.77% | 1.49% |
| 37 | DM | 0.79% | 0.44% | 2.49% | 1.54% | 4.20% |  | 4.81% | 3.36% | 3.21% | 0.22% | 0.69% |  | 2.69% | 0.07% | 4.72% | 0.95% | 0.02% |  | 3.63% | 2.67% | 2.77% | 3.49% | 2.92% | 2.03% |  | 4.34% | 3.57% | 4.56% | 3.67% | 4.78% | 3.35% |  | 4.52% | 4.26% | 4.68% | 3.35% | 4.00% | 1.25% |
| 38 | DM | 3.31% | 1.37% | 3.74% | 1.80% | 3.62% |  | 0.70% | 2.48% | 3.26% | 2.16% | 4.73% |  | 0.52% | 0.59% | 3.07% | 3.38% | 3.96% |  | 3.67% | 3.63% | 3.81% | 3.63% | 2.72% | 3.67% |  | 4.38% | 4.41% | 4.49% | 3.63% | 4.90% | 3.15% |  | 4.59% | 3.73% | 4.10% | 3.76% | 2.41% | 3.30% |
| 39 | DM | 0.04% | 0.00% | 4.29% | 0.80% | 0.82% |  | 3.72% | 3.12% | 3.67% | 4.20% | 4.52% |  | 4.29% | 4.84% | 3.31% | 3.50% | 0.09% |  | 3.54% | 2.47% | 4.38% | 3.72% | 2.27% | 4.41% |  | 4.42% | 4.45% | 4.12% | 3.58% | 4.93% | 1.81% |  | 4.37% | 3.82% | 4.59% | 3.94% | 2.97% | 4.23% |
| 40 | DM | 4.99% | 4.58% | 3.36% | 0.71% | 1.64% |  | 0.53% | 2.73% | 3.81% | 2.07% | 3.70% |  | 3.96% | 5.00% | 4.38% | 4.85% | 5.00% |  | 3.31% | 2.52% | 4.47% | 3.67% | 2.47% | 2.37% |  | 4.30% | 4.26% | 4.49% | 3.81% | 3.22% | 4.97% |  | 4.06% | 4.14% | 4.65% | 3.98% | 1.71% | 3.30% |
| 41 | DM | 3.47% | 4.97% | 3.98% | 0.84% | 1.32% |  | 0.00% | 3.98% | 4.44% | 3.92% | 2.38% |  | 3.25% | 2.73% | 4.13% | 4.95% | 1.56% |  | 3.36% | 4.56% | 3.76% | 3.76% | 4.13% | 2.72% |  | 4.17% | 3.51% | 4.45% | 4.02% | 3.41% | 3.40% |  | 4.33% | 4.61% | 4.10% | 3.35% | 4.33% | 5.00% |
| 42 | DM | 0.61% | 4.24% | 4.61% | 0.67% | 1.52% |  | 4.57% | 4.21% | 2.87% | 4.62% | 3.46% |  | 1.85% | 1.11% | 4.47% | 4.69% | 3.46% |  | 3.45% | 4.69% | 3.63% | 3.26% | 3.31% | 3.93% |  | 4.66% | 3.77% | 4.49% | 3.76% | 4.30% | 3.44% |  | 4.45% | 3.26% | 3.85% | 3.44% | 1.64% | 4.93% |
| 43 | DM | 2.58% | 3.59% | 3.22% | 0.82% | 3.96% |  | 0.13% | 4.99% | 4.90% | 0.88% | 2.16% |  | 4.75% | 1.06% | 3.36% | 4.10% | 5.00% |  | 3.89% | 3.45% | 3.72% | 3.31% | 3.21% | 3.63% |  | 4.26% | 4.00% | 4.42% | 3.90% | 3.83% | 1.91% |  | 4.52% | 4.18% | 4.52% | 3.54% | 3.33% | 3.04% |
| 44 | DM | 0.59% | 0.86% | 2.89% | 1.25% | 3.74% |  | 0.46% | 4.84% | 3.16% | 0.01% | 1.44% |  | 0.04% | 2.22% | 4.50% | 0.92% | 3.74% |  | 3.12% | 3.31% | 3.31% | 3.67% | 3.02% | 4.53% |  | 4.08% | 3.77% | 4.25% | 3.30% | 4.92% | 4.59% |  | 4.72% | 4.26% | 4.48% | 3.76% | 2.55% | 2.72% |
| 45 | DM | 0.08% | 2.57% | 2.67% | 2.01% | 4.29% |  | 2.95% | 3.59% | 3.12% | 4.13% | 2.86% |  | 3.38% | 0.14% | 4.76% | 3.66% | 0.01% |  | 3.89% | 2.82% | 3.63% | 3.49% | 3.72% | 3.63% |  | 4.46% | 4.34% | 4.42% | 3.58% | 4.98% | 4.47% |  | 4.33% | 3.82% | 4.36% | 3.81% | 4.99% | 2.72% |
| 46 | NPDR | 1.71% | 0.38% | 3.69% | 1.47% | 4.73% |  | 1.73% | 3.20% | 4.13% | 4.44% | 4.07% |  | 4.98% | 0.81% | 4.44% | 3.29% | 2.91% |  | 4.93% | 4.74% | 4.89% | 4.09% | 4.53% | 4.44% |  | 4.66% | 4.99% | 4.82% | 4.25% | 3.93% | 4.98% |  | 3.94% | 4.68% | 4.12% | 3.54% | 4.87% | 4.91% |
| 47 | NPDR | 3.54% | 3.73% | 4.10% | 1.73% | 4.69% |  | 3.96% | 2.71% | 4.90% | 1.24% | 0.32% |  | 1.64% | 0.02% | 4.17% | 2.99% | 0.05% |  | 4.95% | 4.89% | 4.69% | 4.93% | 4.09% | 4.69% |  | 4.49% | 4.86% | 4.96% | 5.00% | 4.95% | 4.01% |  | 2.81% | 4.80% | 4.83% | 3.81% | 4.93% | 4.48% |
| 48 | NPDR | 3.79% | 0.28% | 3.29% | 1.89% | 0.57% |  | 4.41% | 0.93% | 4.78% | 0.66% | 0.37% |  | 2.47% | 1.63% | 4.90% | 0.02% | 0.78% |  | 4.98% | 4.47% | 4.05% | 4.69% | 4.59% | 4.24% |  | 3.83% | 4.55% | 4.88% | 4.49% | 4.69% | 4.05% |  | 0.16% | 3.29% | 4.69% | 4.25% | 4.97% | 4.11% |
| 49 | NPDR | 4.69% | 0.16% | 3.06% | 1.52% | 4.80% |  | 3.89% | 1.26% | 4.38% | 4.33% | 0.85% |  | 0.02% | 3.70% | 5.00% | 3.25% | 1.52% |  | 5.00% | 4.72% | 4.72% | 3.72% | 3.72% | 4.38% |  | 4.77% | 4.96% | 4.98% | 3.90% | 4.75% | 4.29% |  | 0.43% | 2.80% | 4.57% | 3.06% | 4.99% | 4.79% |
| 50 | NPDR | 4.96% | 0.33% | 4.48% | 1.25% | 0.06% |  | 0.60% | 0.01% | 3.40% | 3.38% | 4.20% |  | 3.21% | 1.09% | 4.95% | 4.73% | 3.50% |  | 5.00% | 4.87% | 3.40% | 3.36% | 3.31% | 3.63% |  | 4.97% | 3.86% | 5.00% | 4.06% | 4.38% | 4.74% |  | 2.02% | 4.65% | 4.99% | 4.94% | 4.32% | 3.93% |
| 51 | NPDR | 4.64% | 0.00% | 4.70% | 1.03% | 0.17% |  | 4.36% | 1.00% | 3.21% | 4.44% | 1.60% |  | 4.97% | 0.02% | 4.72% | 4.77% | 1.60% |  | 4.59% | 5.00% | 3.21% | 2.72% | 3.49% | 2.82% |  | 4.91% | 4.48% | 4.19% | 4.61% | 4.09% | 5.00% |  | 4.98% | 4.65% | 5.00% | 4.97% | 0.66% | 3.30% |
| 52 | NPDR | 4.66% | 0.03% | 4.27% | 0.91% | 4.73% |  | 0.02% | 4.83% | 3.59% | 0.08% | 3.74% |  | 4.36% | 0.53% | 4.93% | 1.81% | 4.93% |  | 4.86% | 4.09% | 3.67% | 4.50% | 4.99% | 4.41% |  | 4.82% | 4.13% | 4.65% | 4.29% | 4.86% | 4.69% |  | 4.13% | 4.68% | 5.00% | 4.81% | 4.55% | 3.89% |
| 53 | NPDR | 1.83% | 1.03% | 4.42% | 1.52% | 0.02% |  | 2.47% | 2.76% | 4.93% | 0.02% | 0.57% |  | 4.33% | 4.97% | 3.26% | 4.94% | 1.24% |  | 4.98% | 4.38% | 2.77% | 4.09% | 4.76% | 4.98% |  | 5.00% | 2.81% | 4.16% | 4.80% | 4.70% | 4.67% |  | 4.99% | 4.99% | 4.32% | 4.52% | 4.07% | 3.70% |
| 54 | NPDR | 1.29% | 0.46% | 3.48% | 1.20% | 1.60% |  | 0.02% | 4.03% | 3.02% | 0.57% | 1.60% |  | 4.13% | 4.33% | 4.53% | 0.05% | 0.99% |  | 4.69% | 3.76% | 4.44% | 4.44% | 4.82% | 4.41% |  | 0.25% | 4.99% | 4.97% | 3.35% | 4.56% | 3.89% |  | 4.15% | 4.85% | 4.86% | 4.39% | 4.84% | 4.96% |
| 55 | NPDR | 4.71% | 1.68% | 3.25% | 1.12% | 4.98% |  | 3.85% | 1.09% | 2.77% | 4.95% | 1.40% |  | 0.88% | 4.15% | 4.59% | 0.01% | 1.85% |  | 4.44% | 4.76% | 4.78% | 3.26% | 3.45% | 4.27% |  | 1.06% | 0.63% | 4.83% | 4.84% | 4.84% | 3.62% |  | 3.91% | 4.12% | 3.55% | 4.68% | 4.37% | 4.72% |
| 56 | NPDR | 3.79% | 3.92% | 3.32% | 1.24% | 1.24% |  | 3.38% | 4.99% | 2.67% | 0.72% | 0.01% |  | 4.67% | 1.35% | 4.69% | 3.38% | 4.44% |  | 3.36% | 3.21% | 4.94% | 2.77% | 4.57% | 4.51% |  | 2.40% | 1.64% | 5.00% | 4.97% | 4.88% | 2.77% |  | 4.78% | 3.85% | 1.75% | 2.04% | 4.54% | 4.19% |
| 57 | NPDR | 3.54% | 3.44% | 3.69% | 1.89% | 0.03% |  | 4.64% | 4.82% | 3.21% | 0.13% | 0.05% |  | 4.94% | 0.28% | 4.91% | 4.07% | 2.07% |  | 4.76% | 4.82% | 4.82% | 2.97% | 4.87% | 4.82% |  | 2.04% | 4.22% | 4.10% | 4.86% | 4.98% | 4.01% |  | 3.37% | 2.24% | 2.72% | 4.94% | 4.73% | 5.00% |
| 58 | NPDR | 2.00% | 4.71% | 3.82% | 2.19% | 3.92% |  | 0.00% | 3.28% | 3.26% | 0.02% | 0.49% |  | 3.92% | 0.02% | 4.89% | 4.96% | 4.10% |  | 4.97% | 4.97% | 3.63% | 3.21% | 4.72% | 2.82% |  | 0.07% | 4.64% | 1.94% | 4.94% | 5.00% | 3.40% |  | 0.00% | 4.67% | 4.34% | 4.63% | 4.59% | 3.56% |
| 59 | NPDR | 2.40% | 2.76% | 3.37% | 1.26% | 2.38% |  | 3.46% | 4.08% | 3.63% | 0.03% | 0.03% |  | 4.50% | 0.47% | 4.98% | 1.81% | 3.42% |  | 3.98% | 5.00% | 4.80% | 3.72% | 4.78% | 3.36% |  | 4.84% | 4.80% | 4.81% | 3.49% | 3.33% | 4.23% |  | 3.68% | 3.13% | 3.55% | 4.52% | 4.73% | 3.46% |
| 60 | NPDR | 4.66% | 0.46% | 2.92% | 2.16% | 3.29% |  | 4.96% | 0.60% | 4.47% | 3.96% | 3.12% |  | 1.09% | 4.27% | 4.86% | 4.30% | 0.88% |  | 4.51% | 4.90% | 4.99% | 4.13% | 3.16% | 2.27% |  | 4.29% | 4.82% | 4.01% | 3.30% | 4.99% | 4.85% |  | 0.02% | 2.02% | 4.10% | 4.49% | 3.72% | 2.55% |
| 61 | NPDR | 1.80% | 0.75% | 3.39% | 1.75% | 2.38% |  | 0.46% | 0.06% | 4.78% | 4.73% | 1.32% |  | 4.33% | 4.88% | 3.40% | 3.38% | 3.50% |  | 4.84% | 4.72% | 4.98% | 4.90% | 2.52% | 4.98% |  | 4.77% | 4.70% | 3.75% | 3.76% | 4.29% | 3.82% |  | 1.76% | 1.39% | 4.59% | 4.36% | 4.79% | 4.45% |
| 62 | NPDR | 0.25% | 0.36% | 3.74% | 1.20% | 0.09% |  | 1.40% | 3.82% | 4.99% | 0.02% | 1.24% |  | 0.07% | 1.66% | 4.89% | 0.17% | 1.32% |  | 3.17% | 3.02% | 4.95% | 2.82% | 4.47% | 4.35% |  | 4.17% | 4.64% | 4.40% | 3.81% | 4.42% | 4.97% |  | 4.58% | 4.83% | 3.90% | 4.73% | 3.13% | 4.15% |
| 63 | NPDR | 2.82% | 0.05% | 3.42% | 1.12% | 0.14% |  | 3.58% | 2.04% | 4.41% | 0.26% | 4.36% |  | 4.64% | 0.01% | 4.69% | 0.01% | 0.07% |  | 3.67% | 3.67% | 4.82% | 5.00% | 4.87% | 4.90% |  | 5.00% | 4.30% | 3.61% | 3.76% | 3.64% | 4.61% |  | 4.43% | 4.20% | 3.92% | 4.14% | 2.07% | 3.04% |
| 64 | NPDR | 0.51% | 0.29% | 3.82% | 1.14% | 0.01% |  | 0.04% | 3.84% | 4.47% | 0.72% | 0.75% |  | 0.19% | 3.20% | 4.53% | 4.99% | 2.51% |  | 4.05% | 4.24% | 4.51% | 4.98% | 3.07% | 4.87% |  | 4.49% | 4.48% | 3.34% | 4.18% | 3.93% | 4.46% |  | 3.84% | 0.79% | 0.75% | 4.32% | 4.91% | 1.16% |
| 65 | NPDR | 0.46% | 4.87% | 4.92% | 1.30% | 4.13% |  | 4.36% | 3.75% | 4.80% | 3.96% | 4.13% |  | 4.64% | 1.63% | 2.92% | 1.17% | 1.77% |  | 3.21% | 4.78% | 4.80% | 4.47% | 4.91% | 3.68% |  | 4.99% | 4.67% | 4.45% | 3.85% | 2.87% | 4.05% |  | 1.44% | 4.99% | 0.67% | 4.02% | 4.06% | 1.70% |
| 66 | NPDR | 0.01% | 1.65% | 3.37% | 1.47% | 4.03% |  | 4.79% | 2.35% | 4.69% | 3.38% | 4.29% |  | 0.82% | 1.16% | 2.27% | 1.77% | 2.95% |  | 4.94% | 3.63% | 4.67% | 4.31% | 4.99% | 2.27% |  | 0.07% | 4.55% | 4.42% | 3.90% | 4.79% | 4.99% |  | 0.47% | 5.00% | 2.75% | 4.39% | 3.83% | 3.80% |
| 67 | PDR | 3.18% | 4.97% | 5.00% | 2.47% | 4.44% |  | 1.28% | 0.04% | 4.78% | 2.29% | 0.54% |  | 0.13% | 4.98% | 5.00% | 4.85% | 4.33% |  | 4.95% | 4.51% | 4.98% | 4.20% | 4.94% | 4.86% |  | 1.53% | 4.58% | 3.65% | 4.58% | 3.64% | 5.00% |  | 0.16% | 3.89% | 3.60% | 5.00% | 1.79% | 1.75% |
| 68 | PDR | 0.05% | 3.39% | 4.67% | 2.77% | 0.37% |  | 0.01% | 1.77% | 4.74% | 2.91% | 0.02% |  | 3.46% | 4.63% | 4.94% | 4.10% | 1.94% |  | 4.98% | 5.00% | 1.98% | 4.09% | 4.98% | 4.57% |  | 0.96% | 3.77% | 4.15% | 4.55% | 2.94% | 4.86% |  | 1.33% | 3.65% | 2.84% | 4.93% | 0.98% | 2.61% |
| 69 | PDR | 0.01% | 4.65% | 4.89% | 1.98% | 4.91% |  | 0.20% | 0.45% | 4.47% | 2.82% | 0.05% |  | 2.78% | 5.00% | 4.90% | 1.44% | 1.13% |  | 4.98% | 4.80% | 4.89% | 4.44% | 5.00% | 4.95% |  | 1.98% | 3.82% | 4.48% | 4.78% | 4.24% | 4.88% |  | 2.26% | 4.16% | 3.38% | 4.77% | 3.20% | 3.65% |
| 70 | PDR | 3.57% | 4.18% | 4.94% | 1.74% | 3.46% |  | 4.52% | 2.39% | 4.62% | 2.29% | 2.51% |  | 3.70% | 4.61% | 4.78% | 0.08% | 2.69% |  | 4.98% | 5.00% | 4.94% | 3.81% | 4.94% | 4.90% |  | 2.22% | 4.97% | 4.10% | 4.36% | 3.93% | 4.29% |  | 3.94% | 3.40% | 3.55% | 4.95% | 1.93% | 3.98% |
| 71 | PDR | 0.70% | 3.60% | 4.51% | 1.50% | 2.51% |  | 0.46% | 0.93% | 4.90% | 2.38% | 4.95% |  | 4.96% | 3.09% | 4.91% | 1.90% | 1.90% |  | 4.78% | 4.94% | 4.99% | 3.81% | 4.67% | 4.54% |  | 2.40% | 4.82% | 2.85% | 3.85% | 4.34% | 4.05% |  | 2.81% | 3.02% | 3.87% | 5.00% | 2.49% | 4.31% |
| 72 | PDR | 4.53% | 1.54% | 4.44% | 1.74% | 3.38% |  | 1.98% | 0.14% | 4.87% | 3.92% | 4.69% |  | 0.00% | 3.74% | 4.59% | 4.47% | 0.03% |  | 4.51% | 4.76% | 4.76% | 3.67% | 4.94% | 4.31% |  | 3.57% | 4.18% | 4.81% | 4.18% | 2.94% | 4.58% |  | 0.16% | 3.13% | 4.02% | 3.44% | 1.59% | 4.66% |
| 73 | PDR | 0.11% | 2.76% | 4.94% | 3.02% | 1.44% |  | 1.48% | 3.53% | 4.50% | 4.91% | 0.44% |  | 4.38% | 1.76% | 5.00% | 4.98% | 0.69% |  | 2.13% | 4.51% | 4.93% | 3.31% | 4.99% | 4.94% |  | 4.07% | 3.09% | 4.15% | 4.52% | 2.54% | 3.28% |  | 0.50% | 4.32% | 4.34% | 3.30% | 1.09% | 1.70% |
| 74 | PDR | 1.04% | 3.55% | 4.96% | 3.21% | 0.03% |  | 1.48% | 1.82% | 4.97% | 0.82% | 0.07% |  | 1.85% | 4.30% | 4.94% | 0.01% | 2.82% |  | 1.98% | 4.38% | 4.57% | 4.90% | 4.94% | 4.84% |  | 3.67% | 2.81% | 4.62% | 3.85% | 1.30% | 4.09% |  | 1.79% | 4.16% | 4.07% | 3.54% | 0.40% | 3.15% |
| 75 | PDR | 0.00% | 1.85% | 4.67% | 2.82% | 3.58% |  | 3.70% | 5.00% | 4.93% | 3.96% | 0.05% |  | 3.34% | 1.09% | 4.67% | 1.06% | 1.69% |  | 2.58% | 4.02% | 4.31% | 3.31% | 4.82% | 4.76% |  | 4.88% | 3.62% | 4.48% | 0.88% | 0.37% | 4.32% |  | 0.05% | 4.85% | 3.32% | 2.60% | 1.79% | 3.89% |
| 76 | PDR | 4.85% | 3.60% | 4.31% | 2.87% | 4.47% |  | 0.60% | 2.86% | 4.91% | 0.26% | 1.94% |  | 2.25% | 0.17% | 4.02% | 0.20% | 2.78% |  | 4.21% | 3.41% | 4.94% | 2.87% | 4.47% | 3.32% |  | 0.01% | 2.42% | 3.08% | 4.66% | 0.58% | 3.52% |  | 4.98% | 1.39% | 2.66% | 4.93% | 4.02% | 4.06% |
| 77 | PDR | 0.98% | 0.93% | 5.00% | 3.21% | 4.57% |  | 3.85% | 1.67% | 4.24% | 3.46% | 0.37% |  | 1.40% | 4.33% | 2.63% | 0.02% | 0.01% |  | 3.98% | 3.27% | 5.00% | 2.52% | 4.69% | 4.02% |  | 0.44% | 3.20% | 3.48% | 3.35% | 1.80% | 3.89% |  | 5.00% | 3.18% | 4.99% | 4.77% | 3.26% | 0.73% |
| 78 | PDR | 4.96% | 0.03% | 5.00% | 1.69% | 0.07% |  | 1.81% | 0.31% | 3.85% | 4.57% | 1.52% |  | 4.26% | 5.00% | 4.28% | 1.06% | 0.04% |  | 4.10% | 4.78% | 4.02% | 4.74% | 4.21% | 4.28% |  | 2.78% | 1.19% | 4.15% | 4.18% | 3.00% | 2.15% |  | 4.29% | 3.70% | 4.83% | 3.81% | 2.78% | 0.93% |
| 79 | PDR | 1.11% | 0.00% | 4.94% | 3.76% | 0.02% |  | 2.82% | 2.27% | 3.40% | 4.23% | 4.13% |  | 0.95% | 1.00% | 4.94% | 1.81% | 1.44% |  | 0.05% | 4.62% | 3.94% | 4.69% | 3.98% | 4.80% |  | 3.06% | 4.18% | 3.65% | 4.94% | 2.60% | 4.99% |  | 2.81% | 4.99% | 2.72% | 4.55% | 4.85% | 4.48% |
| 80 | PDR | 0.58% | 0.13% | 5.00% | 0.87% | 4.98% |  | 4.10% | 4.88% | 2.52% | 4.57% | 0.05% |  | 2.99% | 0.05% | 3.72% | 0.05% | 4.20% |  | 3.36% | 4.76% | 4.93% | 4.89% | 3.22% | 4.65% |  | 4.84% | 0.48% | 4.10% | 4.36% | 4.29% | 4.89% |  | 1.18% | 4.99% | 0.40% | 4.75% | 4.79% | 4.23% |
| 81 | PDR | 1.48% | 1.70% | 3.72% | 1.88% | 0.78% |  | 4.94% | 4.84% | 2.03% | 3.66% | 3.17% |  | 2.51% | 3.75% | 4.27% | 2.47% | 2.16% |  | 3.67% | 4.67% | 4.97% | 4.76% | 4.51% | 4.94% |  | 4.25% | 4.09% | 4.87% | 4.58% | 3.64% | 4.74% |  | 1.12% | 4.56% | 0.99% | 4.39% | 4.89% | 4.64% |
| 82 | PDR | 2.41% | 4.66% | 4.50% | 2.62% | 4.50% |  | 2.51% | 1.00% | 4.69% | 4.80% | 0.72% |  | 4.73% | 1.82% | 4.94% | 2.51% | 4.91% |  | 4.41% | 3.98% | 4.78% | 4.82% | 4.78% | 4.99% |  | 4.90% | 4.61% | 2.30% | 4.49% | 1.93% | 4.99% |  | 0.02% | 3.80% | 4.16% | 4.58% | 4.93% | 4.81% |
| 83 | PDR | 0.94% | 1.90% | 4.53% | 2.08% | 0.69% |  | 0.19% | 1.58% | 4.28% | 1.85% | 3.17% |  | 0.44% | 2.35% | 3.26% | 1.86% | 0.05% |  | 2.47% | 4.65% | 4.89% | 4.62% | 4.86% | 4.89% |  | 3.62% | 4.88% | 2.00% | 4.06% | 1.93% | 1.18% |  | 3.62% | 4.71% | 5.00% | 4.25% | 4.95% | 4.31% |
| 84 | PDR | 0.01% | 0.73% | 4.69% | 2.67% | 1.73% |  | 1.24% | 4.97% | 4.44% | 0.05% | 3.54% |  | 4.82% | 1.43% | 2.92% | 0.01% | 2.60% |  | 2.42% | 3.77% | 4.95% | 4.95% | 5.00% | 4.76% |  | 2.10% | 4.80% | 1.43% | 4.69% | 4.75% | 4.97% |  | 1.79% | 1.91% | 4.53% | 3.98% | 4.94% | 1.70% |
| 85 | PDR | 4.60% | 0.03% | 3.94% | 2.67% | 2.03% |  | 1.64% | 1.13% | 4.98% | 1.98% | 3.25% |  | 1.73% | 4.79% | 4.69% | 4.95% | 0.44% |  | 4.93% | 4.72% | 4.41% | 4.20% | 4.82% | 4.98% |  | 4.46% | 4.55% | 3.54% | 4.76% | 3.20% | 4.88% |  | 0.20% | 3.02% | 4.93% | 4.66% | 4.70% | 2.01% |
| 86 | PDR | 2.36% | 0.63% | 4.59% | 3.49% | 2.82% |  | 3.85% | 1.34% | 4.94% | 4.71% | 4.30% |  | 4.36% | 4.93% | 4.67% | 1.77% | 2.99% |  | 4.82% | 4.69% | 4.74% | 4.47% | 4.93% | 2.58% |  | 4.84% | 3.91% | 3.96% | 4.52% | 3.14% | 4.69% |  | 4.29% | 3.18% | 3.32% | 4.36% | 4.95% | 0.42% |
| 87 | PDR | 1.08% | 3.44% | 4.94% | 3.67% | 4.73% |  | 4.98% | 0.17% | 4.86% | 0.01% | 2.60% |  | 1.17% | 4.95% | 4.35% | 3.78% | 4.35% |  | 4.90% | 4.97% | 4.24% | 4.20% | 4.69% | 2.98% |  | 3.48% | 4.37% | 4.98% | 4.25% | 4.98% | 4.43% |  | 4.99% | 3.13% | 4.07% | 4.21% | 5.00% | 4.99% |
| 88 | PDR | 1.11% | 2.76% | 5.00% | 2.17% | 3.29% |  | 3.58% | 5.00% | 4.51% | 4.69% | 4.89% |  | 3.12% | 0.89% | 3.90% | 0.72% | 0.06% |  | 0.39% | 4.90% | 3.76% | 3.85% | 3.63% | 4.62% |  | 4.92% | 3.46% | 5.00% | 4.18% | 0.00% | 2.77% |  | 0.16% | 4.39% | 0.23% | 3.81% | 4.99% | 1.20% |
| 89 | PDR | 4.95% | 1.68% | 3.36% | 1.37% | 2.78% |  | 0.00% | 4.99% | 4.62% | 4.73% | 4.73% |  | 0.00% | 0.85% | 4.59% | 1.85% | 3.29% |  | 3.17% | 4.99% | 5.00% | 3.93% | 3.26% | 3.03% |  | 3.37% | 4.98% | 4.86% | 4.29% | 1.80% | 4.17% |  | 4.90% | 4.56% | 0.16% | 4.52% | 4.79% | 2.23% |
| 90 | PDR | 2.00% | 0.12% | 4.98% | 0.69% | 2.20% |  | 5.00% | 1.90% | 4.96% | 3.38% | 1.06% |  | 0.66% | 1.95% | 2.23% | 0.63% | 2.60% |  | 2.97% | 4.72% | 4.13% | 4.09% | 4.90% | 2.47% |  | 2.40% | 4.99% | 4.28% | 4.61% | 4.70% | 3.18% |  | 3.91% | 4.65% | 4.57% | 4.45% | 4.73% | 4.23% |
| 91 | PDR | 4.99% | 0.18% | 4.78% | 1.32% | 4.94% |  | 4.13% | 5.00% | 2.73% | 3.17% | 0.05% |  | 4.96% | 2.80% | 2.87% | 0.09% | 0.99% |  | 3.21% | 5.00% | 3.63% | 3.02% | 4.10% | 2.63% |  | 3.23% | 4.34% | 1.54% | 4.80% | 4.98% | 4.29% |  | 4.99% | 3.75% | 4.07% | 3.85% | 4.82% | 4.94% |
